# Mitochondrial quality control and its role in osteoporosis

**DOI:** 10.3389/fendo.2023.1077058

**Published:** 2023-01-30

**Authors:** Chunlu Yan, Yao Shi, Lingqing Yuan, Donghui Lv, Bai Sun, Jiayu Wang, Xiyan Liu, Fangyu An

**Affiliations:** ^1^ School of Traditional Chinese and Western Medicine, Gansu University of Chinese Medicine, Lanzhou, Gansu, China; ^2^ Research Center of Traditional Chinese Medicine of Gansu, Gansu University of Chinese Medicine, Lanzhou, Gansu, China; ^3^ School of Basic Medicine, Gansu University of Chinese Medicine, Lanzhou, Gansu, China; ^4^ Internal Medicine, Northwestern University, Xian, Shanxi, China; ^5^ Teaching Experiment Training Center, Gansu University of Chinese Medicine, Lanzhou, Gansu, China

**Keywords:** osteoporosis, mitochondrial biogenesis, mitochondrial autophagy, targeted therapy, mitochondrial fusion and fission

## Abstract

Mitochondria are important organelles that provide cellular energy and play a vital role in cell differentiation and apoptosis. Osteoporosis is a chronic metabolic bone disease mainly caused by an imbalance in osteoblast and osteoclast activity. Under physiological conditions, mitochondria regulate the balance between osteogenesis and osteoclast activity and maintain bone homeostasis. Under pathological conditions, mitochondrial dysfunction alters this balance; this disruption is important in the pathogenesis of osteoporosis. Because of the role of mitochondrial dysfunction in osteoporosis, mitochondrial function can be targeted therapeutically in osteoporosis-related diseases. This article reviews different aspects of the pathological mechanism of mitochondrial dysfunction in osteoporosis, including mitochondrial fusion and fission, mitochondrial biogenesis, and mitophagy, and highlights targeted therapy of mitochondria in osteoporosis (diabetes induced osteoporosis and postmenopausal osteoporosis) to provide novel targets and prevention strategies for the prevention and treatment of osteoporosis and other chronic bone diseases.

## 1 Introduction

Osteoporosis (OP) is a metabolic disease in which the rate of bone resorption is greater than that of bone formation, resulting in systemic loss of bone mass and changes in bone microstructure (1). Approximately 200 million people worldwide suffer from OP (2). A study in 2019 showed that the prevalence of OP in men and women over 50 years old in China was as high as 6.46% and 29.13%, respectively (3). Reducing the incidence and elucidating the pathogenesis of OP has become an urgent public health issue. In recent years, numerous studies have shown that mitochondrial dysfunction in both osteoblasts and osteoclasts is involved in the progression of OP (4).

Mitochondria, a bilayer membrane organelle present in eukaryotic cells, produce adenosine triphosphate (ATP) through oxidative phosphorylation, providing cellular energy. Mitochondria also play an important role in cell differentiation, apoptosis, and autophagy (5). Under physiological conditions, cells are co-regulated by mitochondrial fusion and fission, mitochondrial biogenesis, and mitophagy, forming a mitochondrial network that influences mitochondrial quality control to maintain mitochondrial homeostasis and ensure normal cellular function. By studying 45 carriers, Langdahl et al. showed that the mitochondrial point mutation m.3243A>G was associated with bone mass, suggesting mitochondrial dysfunction may lead to premature bone aging (6). When mitochondrial function is abnormal, mitochondrial homeostasis is disrupted, leading to intracellular disorder or cellular dysfunction, destroying the balance between osteogenesis and osteoclast activity, ultimately, affecting the occurrence and development of OP. This article reviews different aspects of the pathological mechanism of mitochondrial dysfunction in osteoporosis, including mitochondrial fusion and fission, mitochondrial biogenesis and mitophagy, and highlights targeted therapy of mitochondria in osteoporosis to provide novel targets and prevention strategies for the prevention and treatment of osteoporosis (diabetes induced osteoporosis and postmenopausal osteoporosis) and other chronic bone diseases.

## 2 The pathological role of mitochondria in osteoporosis

### 2.1 Mitochondrial dynamics and osteoporosis

Under physiological conditions, mitochondria provide cellular energy and regulate calcium homeostasis, signaling, and apoptosis by fission and fusion (7). The balance between mitochondrial fission and fusion is essential to maintain mitochondrial quality and function, which are important for normal cellular activity. Recent studies have shown that the main protein regulating mitochondrial fission is dynamic-related protein 1 (DRP1), which is present primarily in the cytoplasm. DRP1 is recruited to mitochondria and oligomerized by binding receptors such as mitochondrial fission factor (Mff), mitochondrial fission protein 1 (Fis1), mitochondrial dynamics protein of 49 kDa (MiD49), and mitochondrial dynamics protein of 51 kDa (MiD51), resulting in GTPase-mediated GTP hydrolysis and rupture of the inner and outer mitochondrial membrane and mitochondrial fission (8). Thus, Drp1 is a key mediator of mitochondrial dynamics; overexpression of Drp1 induces excessive mitochondrial division (9). Mitofusion 1/2 (Mfn1/2) and optic atrophy 1 (OPA1) are involved in mitochondrial fusion. Mfn1/2 is located in the outer mitochondrial membrane and mediates mitochondrial outer membrane fusion, while OPA1 is involved in mitochondrial inner membrane fusion and maintains the integrity of mitochondrial crest structure and the inner membrane (10).

**Figure 1:**
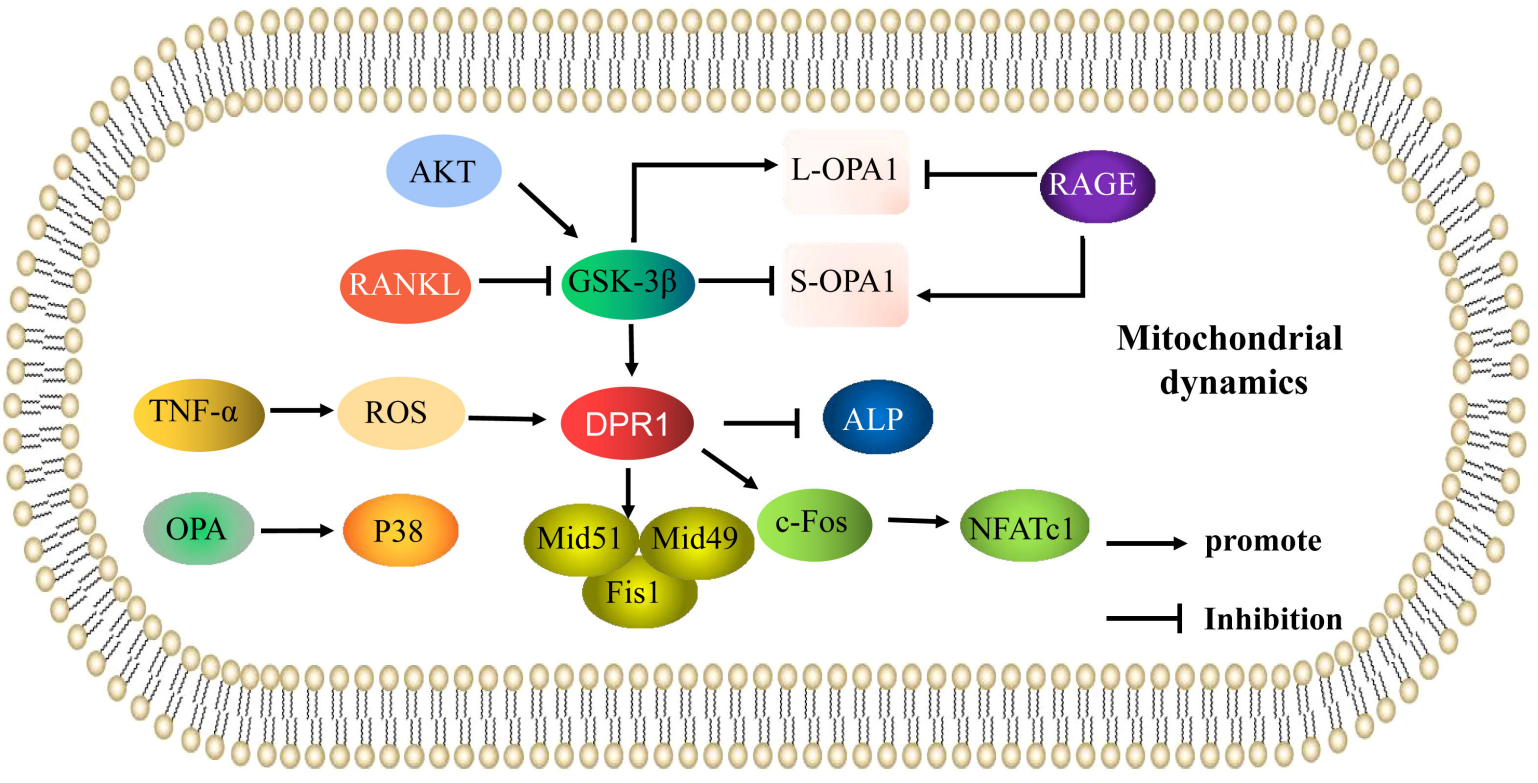
Mitochondrial dynamics are involved in the mechanisms of the regulation of osteoporosis. Mechanisms of mitochondrial dynamics (mitochondrial fusion and fission) that regulate osteoblasts and osteoclasts in osteoporosis. The arrows indicate activation and the inhibitory arrows indicate inhibition.

When mitochondrial fusion and fission are abnormal, osteoblast and osteoclast activity is altered, and the occurrence and development of OP is accelerated. Under oxidative stress, expression of DRP1 and its phosphorylation is increased in osteoblasts, and mitochondria were shown to be fragmented, malformed, and vesicular (11). Blocking DRP1 with pharmacological inhibitors or gene knockout inhibited reactive oxygen species (ROS) production, and increased mitochondrial length and density, alkaline phosphatase (ALP) activity, and bone nodule formation. Zhang (12) demonstrated that tumor necrosis factor (TNF)-α can induce a high level of mitochondrial ROS accumulation, and upregulation of DRP1 triggered a collapse of mitochondrial membrane potential, causing mitochondria to vesiculate and fragment, resulting in a reduction in mitochondrial function and inhibition of osteoblast activity. These studies indicated that oxidative stress induces osteoblast dysfunction through DRP1-mediated mitochondrial hyperdivision. DRP1-mediated mitochondrial hyperdivision is also involved in the occurrence of OP by regulating osteoclasts. Receptor activator of NF-κB ligand (RANKL) has been reported to regulate expression of DRP1 and its receptor proteins Fis1, Mid49, and Mid51. Furthermore, a glycogen synthase kinase-3β (GSK-3β) inhibitor has been shown to increase DRP1 expression and promote osteoclast differentiation. These results suggest the RANKL/GSK-3β/DRP1 axis can promote osteoclast differentiation. DRP1 has been shown to increase osteoclast differentiation by promoting the c-fos/NFATc1 axis, while downregulation of DRP1 significantly reduced expression of c-fos and nuclear factor 1 of activated T-cells (NFATc1), inhibited osteoclast differentiation, prevented bone loss, and played a protective role in a postmenopausal OP mouse model (13). This suggests that mitochondrial dysfunction may be the underlying pathophysiological mechanism of OP in postmenopausal women (14). Ballard (15) showed that knockdown of Mfn1 and Mfn2 in osteoclasts increased bone mass in female mice, and Mfn2 not only promoted mitochondrial fusion (16) but also activated NFATc1 expression (17). However, whether Mfn2-induced impairment of mitochondrial fusion plays a role in osteoclast formation has not yet been investigated. These results suggest an imbalance in mitochondrial kinetics caused by excessive fission and fusion in osteoblasts and osteoclasts may be important in the occurrence and development of OP (**Figure 1** **)**.

Mitochondrial dysfunction affects osteoblast function and is a key mechanism underlying oxidative stress-induced osteoblast apoptosis (18). Several studies have shown that OPA-induced mitochondrial kinetic abnormalities play an important role in apoptosis (19). Increased OPA expression activated p38 signaling, resulting in decreased mitochondrial ATP production and increased bone marrow cell apoptosis in a radiation-induced osteoporosis model. By contrast, the p38 signaling pathway inhibitor U0126 promoted mitochondrial ATP production and inhibited bone marrow cell apoptosis in OP mice by inhibiting OPA expression and p38 signaling (20). Thus, OPA can induce mitochondrial kinetic dysfunction, promote cell apoptosis, and accelerate the occurrence of OP by activating p38 signaling. OPA1 exists in two forms, a long form (L-OPA1) and short form (S-OPA1). L-OPA1 mediates mitochondrial fusion, while S-OPA1 inhibits mitochondrial fusion; Maintaining a balance between L-OPA1 and S-OPA1 is important for normal mitochondrial function (21). Hydrogen peroxide has been shown to promote rapid cleavage of L-OPA1 into S-OPA1, increased mitochondrial ROS (mtROS) production, significantly decreased mitochondrial respiratory chain complex activity, mitochondrial membrane potential, and ATP production, abnormal mitochondrial morphology, and further changes in mitochondrial dynamics and osteoblast apoptosis. Transfection of MC3T3-E1 cells with small interfering RNA (siRNA) targeting OPA1 inhibited osteoblast apoptosis, demonstrating that cleavage of the mitochondrial dysfunction-related protein OPA1 promotes osteoblast apoptosis. The phosphoinositide 3-kinase (PI3K) inhibitor LY294002 was further shown to increase S-OPA1 and decrease L-OPA1 levels, while the GSK-3β inhibitor TDZD-8 increased L-OPA1 and decreased S-OPA1 levels. Thus, oxidative stress induced OPA1 cleavage by directly regulating protein kinase B(AKT)/GSK-3β signaling, promoting osteoblast apoptosis (22). Mao (23) showed advanced glycation end products (AGEs) significantly increased expression of Fis1 and S-OPA1, reduced expression of L-OPA1, increased production of mtROS, and markedly decreased mitochondrial membrane potential, resulting in fragmented, misshapen, and bleb-like mitochondria and osteoblast MC3T3-E1 apoptosis. However, as a receptor of AGEs, an inhibitor of RAGE significantly decreased Fis1 and S-OPA1 expression, increased the level of L-OPA1, reduced mtROS production, and significantly increased mitochondrial membrane potential and ATP generation; mitochondrial morphology was normal, thus reducing osteoblast apoptosis. RAGE protects against diabetic OP by regulating the balance between L-OPA1 and S-OPA1 to maintain mitochondrial kinetic function and inhibit AGE-induced osteoblast apoptosis. Therefore, regulation of the L-OPA1 and S-OPA1 balance is important in maintaining mitochondrial function and regulation of osteoblast apoptosis in OP (**Figure 1**). These results suggest that regulating the balance of L-OPA1 and S-OPA1 in osteoblasts to maintain mitochondrial function may be an important strategy in the treatment of OP.

### 2.2 Mitochondrial biogenesis and osteoporosis

Mitochondrial biogenesis refers to the formation of new mitochondria, primarily composed of nuclear genes and mitochondrial DNA (mtDNA). The mitochondrial respiratory and ATP synthase complexes produce ATP, the catalytic core subunit of the mitochondrial respiratory complex and ATP synthase are encoded in mtDNA (24). Therefore, when more ATP is required during stem cell differentiation, mitochondrial biogenesis is upregulated, resulting in increased mitochondrial mass and mtDNA content. Recent studies have shown that peroxisome proliferator-activated receptor-gamma coactivator (PGC)-1α is a major regulator of mitochondrial biogenesis; it can promote nuclear and mtDNA transcription to regulate mitochondrial biogenesis (25). PGC-1α is closely related to mitochondrial respiration regulation and ATP synthesis, which can promote mitochondrial biosynthesis and reduce mitochondrial damage (26). Silencing information regulator 1/3 (SIRT1/3), nuclear respiratory factor 1/2 (Nrf1/2), estrogen related receptor α (ERRα), and other molecules have been shown to form a network with PGC-1α in mitochondrial biogenesis in osteoblasts in OP.

Mitochondrial mass can be increased with osteogenic differentiation, resveratrol has been shown to enhance mtDNA content and mitochondrial quality in a time-dose dependent manner (27). Therefore, as a SIRT1 activator (28), resveratrol can promote mitochondrial biogenesis during osteogenic differentiation. Previous studies have shown that SIRT1 promotes mitochondrial biogenesis through deacetylation of its target protein PGC-1α (29). However, mitochondrial deacetylases and their substrates are associated with mtDNA nucleoids and mitochondrial transcription factor A (TFAM), a key mitochondrial transcription factor and mtDNA copy number regulator (30). These findings suggest that SIRT1 and PGC-1α may also affect mitochondrial function through direct regulation of mitochondrial transcription. Ma (31) showed that resveratrol activated PGC-1α by upregulation of SIRT1 expression, increased the generation of mitochondrial ATP, increased mitochondrial membrane potential, decreased mtROS, increased mitochondrial biogenesis, significantly increased the levels of ALP, osteocalcin (OCN), osteopontin (OPN), and runt-related transcription factor 2 (Runx2), and promoted osteogenic differentiation in MC3T3-E1 cells. Therefore, resveratrol effectively improves mitochondrial biogenesis and promotes osteoblast differentiation *via* a process closely related to that involving SIRT1 and PCG-1α. Overall, SIRT1 promotes mitochondrial biogenesis through deacetylation of its target protein PGC-1α, which may be important for osteogenic differentiation in OP. Nrf2 also regulates mitochondrial function (32). PGC-1α, as a major regulator of mitochondrial biogenesis, co-activates transcription of Nrf1/2, co-regulating the expression of nuclear encoded mitochondrial genes, such as TFAM, cytochrome C, and the mitochondrial complex, which together regulate mitochondrial biogenesis (33). Increased mitochondrial biogenesis can raise Nrf2 activity and ROS levels during osteocytogenesis, and Nrf2 activity promoted osteocytic specification through transcriptional activation of osteocyte-specific genes (34). Treatment of osteoblasts with sciadopitysin increased the levels of SIRT1, PGC-1, NRF-1, and TFAM, and enhanced mitochondrial biogenic activity. These results suggest that sciadopitysin-induced factors enhance the biogenic activity of mitochondria and slow down the occurrence of diabetic bone disease (35). Sodium butyrate (NaB) is produced by fermentation of intestinal microbiota in the large intestine. *In vitro* experiments showed NaB could increase Nrf2/GSK-3β signaling, promote expression of PGC-1α and TFAM, inhibit hydrogen peroxide-induced oxidative damage in MC3T3-E1 cells, and improve antioxidant enzyme activity and ATP production. Reducing ROS levels and promoting osteoblast mineralization and differentiation protected bone microstructure and calcium homeostasis and activated bone metabolism. NaB was shown to promote Nrf2/GSK-3β signaling and mitochondrial function, and improve bone loss (36). Overall, the loop formed by SIRT1-PGC-1α-Nrf1/2 may regulate mitochondrial biogenesis in osteoblasts; its mechanism in the pathogenesis of OP will require further exploration. Li (37) showed that angiotensin II reduced protein levels of SIRT1 and peracetylate forkhead box protein O3 a (FoxO3a), thereby reducing expression and enzyme activity of manganese superoxide dismutase 2 (SOD2) and catalase and inducing mitochondrial oxidative stress and mtDNA damage in osteoblasts. Angiotensin II has been shown to induce mitochondrial oxidative stress and mtDNA damage in osteoblasts by inhibiting the SIRT1-FoxO3a-SOD2 axis, thus accelerating development of OP. Other studies have shown that SIRT1 deletion can increase FoxO acetylation and reduce the levels of FoxO and its target gene heme oxygenase 1 (HO-1), while SIRT1 activation can increase the levels of FoxO and HO-1 and reduce ATP production and mitochondrial activity, thereby inhibiting osteoclast generation and playing an anti-osteoporosis role (38). Thus, SIRT1 can regulate the imbalance between osteogenesis and osteoclast activity and is an important participant in the role of mitochondria in OP.

**Figure 2:**
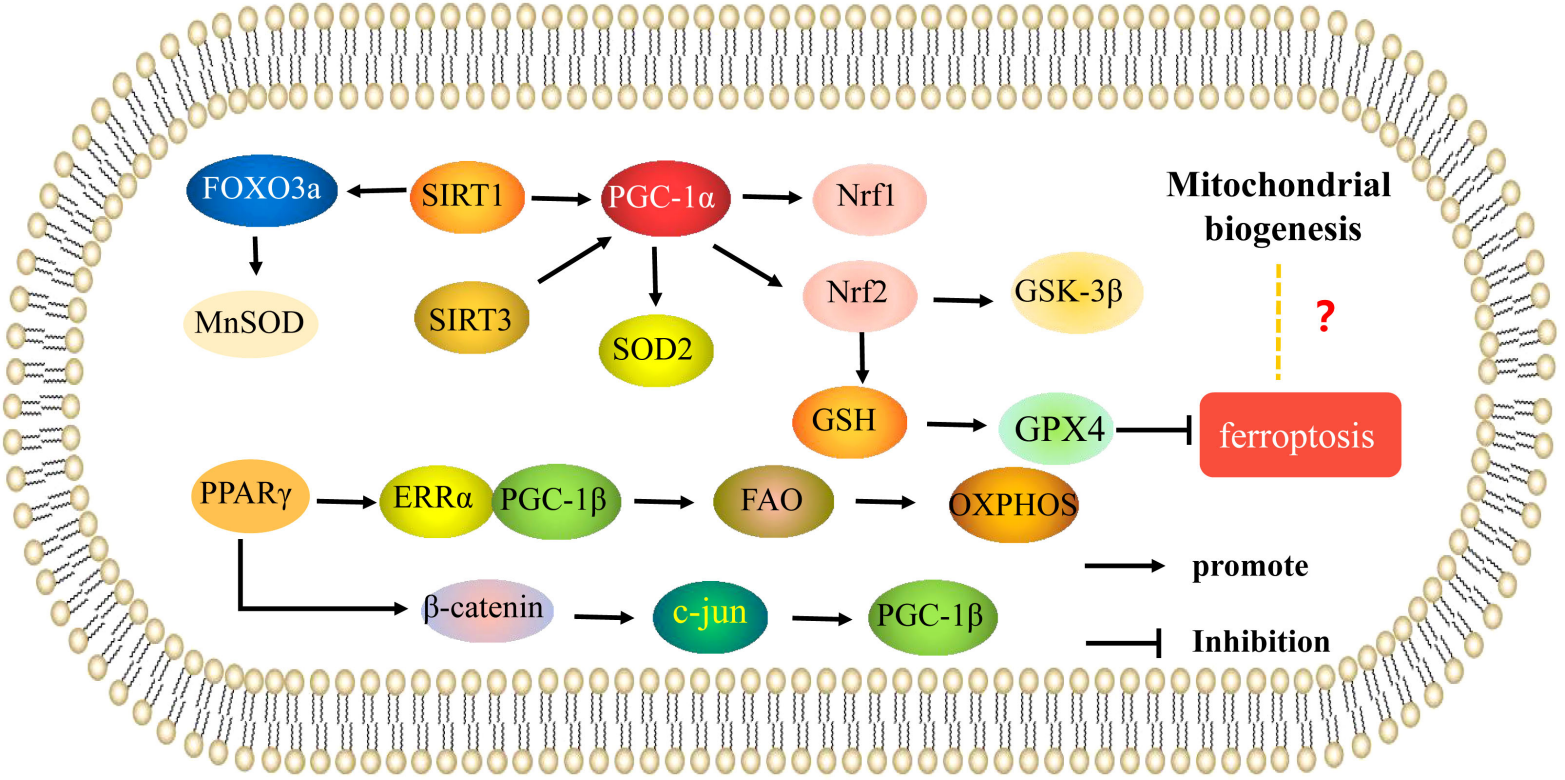
Mitochondrial biogenesis is involved in the mechanisms of regulation of osteoporosis. Mechanisms of mitochondrial biogenesis that regulate osteoblasts and osteoclasts in osteoporosis. The arrows indicate activation and the inhibitory arrows indicate inhibition.

SIRT3 is also important in the role of mitochondria in OP. Ding (39) showed that knockdown of SIRT3 could significantly reduce oxygen consumption, mitochondrial membrane potential, and mitochondrial density, but mitochondrial size increased, expression of Nrf1, TFAM, and SOD2 decreased, and ROS levels increased; activities of complexes I, II, III, IV, and V were also decreased. PGC-1ɑ protein expression was significantly decreased, and expression of Runx2, collagen type 1 alpha1(COL1A1), and osteocalcin were decreased in MC3T3-E1 osteoblasts while overexpression of PGC-1ɑ blocked the reduction in SOD2 expression, increased ROS levels, and decreased mitochondrial function and biosynthesis induced by SIRT3 knockdown, improving osteogenesis. The SIRT3 deacetylase has been shown to play a regulatory role by interacting with PGC-1α. Knockdown of SIRT3 reduced PGC-1α protein stability, binding of PGC-1ɑ to the SOD2 promoter, and mitochondrial biosynthesis. Therefore, regulation of mitochondrial function and biogenesis by SIRT3 *via* PGC-1α-SOD2 signaling affects osteogenic differentiation. The results of Gao et al. are consistent with these results; this study confirmed that SIRT3 knockdown induced decreased SOD2 expression, mitochondrial complex subunits expression, ATP production, and mitochondrial DNA copy number, which increased mitochondrial dysfunction and inhibited osteogenic differentiation. It was further shown that expression of SIRT3 increased during osteogenic differentiation, while K68 acetylation and SOD2 acetylation decreased, indicating that overexpression of SIRT3 enhanced SOD2 activity *via* deacetylation of K68. SIRT3 knockdown in animals reduced SOD2 expression, inhibited mitochondrial biogenesis, reduced expression of Runx2, osterix (OSX), and ALP, inhibited osteogenic differentiation, and reduced bone mass, while SIRT3 overexpression improved mitochondrial biogenesis and promoted osteogenic differentiation. Thus, *in vitro* and *in vivo* experiments have shown that SIRT3 deacetylates SOD2 at K68 in osteoblasts and regulates osteogenic differentiation and bone formation by affecting mitochondrial function (40). Li (41) showed that SIRT3 can deacetylate SOD2 to promote SOD2 activity, inhibit mitochondrial oxidative stress and mtDNA damage, and promote osteoblast function. Thus, SIRT3 is involved in osteoblast differentiation as well as osteoclast differentiation *via* regulation of mitochondrial function. SIRT3 may also play a key role in estrogen deficiency-induced bone loss. Knockdown of SIRT3 significantly reduced mtDNA content, ATP levels, mRNA expression of the mitochondrial biogenesis markers PGC-1α, mitochondrial transcription factor A (mtTFA), and SOD2, inhibited osteoclast differentiation and mitochondrial biogenesis, and increased trabecular bone mass of osteoclasts in female mice (42). These results suggest that SIRT3 and its downstream effectors may be potential therapeutic targets for treatment of postmenopausal OP. Thus, SIRT3/PGC-1α regulates bone metabolism *via* mitochondrial biogenesis. In addition to PGC-1α, PGC-1β also regulates energy metabolism by stimulating mitochondrial biogenesis. Knockdown of PGC-1β has been shown to inhibit osteoclast differentiation and mitochondrial biogenesis *in vitro*, and PGC-1β deletion in mice resulted in increased bone mass (43). Peroxisome proliferators-activated receptors (PPARγ) can indirectly induce PGC-1β expression by downregulating β-catenin protein levels and inhibiting c-Jun expression, thereby promoting osteoclast differentiation. On the other hand, PPARγ can also induce ERRα expression and coordinate with PGC-1β to induce mitochondrial genes involved in fatty acid β-oxidation (β-FAO) and oxidative phosphorylation (OXPHOS) to enhance mitochondrial biogenesis and promote osteoclast differentiation and function (44) (**Figure 2**). Overall, current research on the involvement of mitochondrial biogenesis in the regulation of osteoporosis is mostly related to the mitochondrial biogenesis induced by PGC-1α/β expression and activity.

The gut microbiota has also been recognized as an important factor in many physiological and pathological processes in the host, and an increasing number of studies have shown that the gut microbiota plays a role in bone metabolism and the pathogenesis of OP (45). Through *in vivo* experiments, we found that expression of osteoclast biomarkers such as C-terminal peptide of type 1 collagen (CTX-1), tartrate-resistant acid phosphase (TRAP), c-fos, matrix metalloproteinase-9 (MMP-9), NFATc1, and recombinant cathepsin K (CTSK) was significantly increased in conventional ovariectomized mice, while there was no significant change in sterile ovariectomized mice. Firmicutes and Bacteroidetes were determined to affect the synthesis of glutathione by regulating the catalytic subunit of glutamate-cysteine ligase, a key enzyme involved in glutathione synthesis, and to inhibit mitochondrial biogenesis and ROS accumulation through the camp-responsive element binding pathway, ultimately inhibiting osteoclast differentiation (46). These results indicate that the intestinal flora Firmicutes and Bacteroidetes are important in regulation of mitochondrial biogenesis and osteoclast differentiation. Abnormal osteogenic adipogenic differentiation is also important in the pathogenesis of OP. Mitochondrial biosynthesis and oxygen consumption are significantly increased during adipogenic differentiation, and a reduction in mitochondrial respiration through hypoxia, inhibition of the mitochondrial electron transport chain, and TFAM gene knockout significantly inhibits adipogenic differentiation of bone marrow mesenchymal stem cells (47). Therefore, mitochondrial biosynthesis also regulates adipogenic differentiation, although the specific mechanism is unclear. Targeting mitochondrial biosynthesis may be a new direction for the treatment of OP *via* regulation of adipogenic differentiation.

### 2.3 Mitophagy and osteoporosis

Mitophagy refers to the highly selective degradation and removal of senescent or damaged mitochondria by cells through autophagy to maintain mitochondrial homeostasis and ensure the stability of the intracellular environment (48). The Pten-induced putative kinase 1 (PINK1)/Parkinson disease-related gene (Parkin) pathway is an important pathway in mitophagy (49). PINK1 is a serine kinase that can accumulate in the outer mitochondrial membrane (50), where it promotes the translocation of Parkin to the mitochondrial surface, resulting in the ubiquitination of mitochondrial proteins, aggregation of p62 and specific binding of LC3, thus promoting recruitment of the autophagosome membrane (51) and initiating clearance of damaged mitochondria (52). When mitochondrial membranes depolarize, the PINK1/Parkin pathway is activated to clear damaged mitochondria. Under normal circumstances, PINK1 expression is low outside the mitochondrial membrane, and is mostly transported to the inner mitochondrial membrane by the membrane transporter TOM/TIM complex for degradation. After mitochondrial damage, ROS accumulation and decreased potential outside the membrane leads to loss of potential gradient inside and outside the outer membrane. PINK1 cannot enter the inner membrane and accumulates outside the mitochondrial membrane. In recent years, PINK-Parkin signaling has been found to play an important role in mitophagy impairment in osteoblasts and osteoclasts in OP.

Lee (53) found that PINK1 expression was reduced in OP patients. Bone mass was significantly reduced in an ovariectomized mouse model with PINK1 gene deficiency. *In vitro* experiments showed that expression of PINK1 was increased in osteoblasts during normal osteogenic differentiation, while expression levels of osteogenic markers ALP, bone sialoprotein (BSP), OCN, and OPN were significantly decreased in osteoblasts with low PINK1 expression. There were also more fragmented, abnormal mitochondria. In addition, downregulation of PINK1 induced accumulation of intracellular ROS and mitochondrial superoxide. Thus, mitochondrial dysfunction caused by PINK1 deficiency may be key in preventing osteoblast differentiation and promoting bone mass loss. Zhao (54) found that expression of Prader-Willi/Angelman syndrome region protein 2 (NIPA2) was decreased in bone tissue and osteoblasts in a type 2 diabetic osteoporosis mouse model. Cell experiments showed that overexpression of NIPA2 decreased the expression of LC3-II, PINK1, and Parkin by increasing Mg^2+^ influx; it also inhibited mitophagy, and promoted osteogenic function. Overexpression of NIPA2 was further shown to reduce mitochondrial membrane potential (MMP) and increase osteogenic function by downregulating PGC-1α, promoting expression of FoxO3a, activating BIM, and increasing mitochondrial membrane permeability. Therefore, NIPA2 regulates PINK1/Parkin-mediated mitophagy in osteoblasts through PGC-1α/FoxO3a/MMP signaling. These results indicate that PINK1/Parkin-mediated mitophagy regulation of osteoblasts is a key pathway affecting OP. Wen (55) showed increased acetylation of PINK1 and decreased levels of Bnip3 and Nix in osteoclasts isolated from SIRT3-deficient mice, suggesting SIRT3 promotes osteoclast differentiation and formation, in part, by stimulating mitophagy. These results suggest that PINK1-mediated mitophagy also affects osteoclast differentiation. Other studies have shown that Nrf1 can directly bind the promoter regions of PINK1 and Parkin genes to regulate their expression, and downregulation of Nrf1 inhibits PINK1/Parkin-dependent mitophagy by reducing the expression of PINK1 and Parkin (56). However, Nrf1 can lead to ROS accumulation, regulating the nuclear factor-κB (NF-κB) signaling pathway and increasing expression of NFATc1 to enhance osteoclast differentiation (57). Meanwhile, Nrf1 can also reduce osteoblast differentiation by reducing OSX expression (58). Based on this, we hypothesized that Nrf1 can regulate the balance of osteogenic and osteoclastic differentiation by regulating PINK1/Parkin-dependent mitophagy, which is an important mechanism affecting OP.

Mitochondrial ferritin (FtMt) can also induce mitophagy in osteoblasts. FtMt stores iron ions and intercepts toxic iron ions in mitochondria, and is a key protein in the prevention of iron-dependent cell damage such as ferroptosis. Both ferroptosis and FtMt expression were observed in bone tissue of a type 2 diabetic osteoporosis rat model. Overexpression of FtMt under high glucose conditions reduced ferroptosis and ROS content in osteoblasts, while silencing FtMt induced mitophagy through the ROS/PINK1/Parkin pathway. Activation of mitochondria with the mitophagy agonist carboacylcyanom-chlorobenzene hydrazide (CCCP) increased ferroptosis and ROS expression (59). Thus, reducing FtMt promotes osteoblast ferroptosis by oxidative stress, induced by excess ferrous ions, activating ROS/PINK1/Parkin signaling and enhancing mitophagy in osteoblasts, indicating a positive correlation between mitophagy and ferroptosis. However, activation of Nrf2 has also been shown to increase mitophagy (60); Nrf2 can significantly increase expression of solute carrier protein 7 family member 11 (SLC7A11) to regulate glutathione peroxidase 4 (GPX4) (61) or activate the glutathione (GSH)-GPX4 axis to inhibit ferroptosis (62). Nrf2 also regulates osteogenic differentiation and osteoclast differentiation and participates in bone metabolism, indicating that Nrf2-mediated mitophagy and ferric death are negatively correlated, and may constitute a new mechanism for regulating bone metabolism. However, the specific mechanism underlying mitophagy and ferric death will require further investigation.

**Figure 3:**
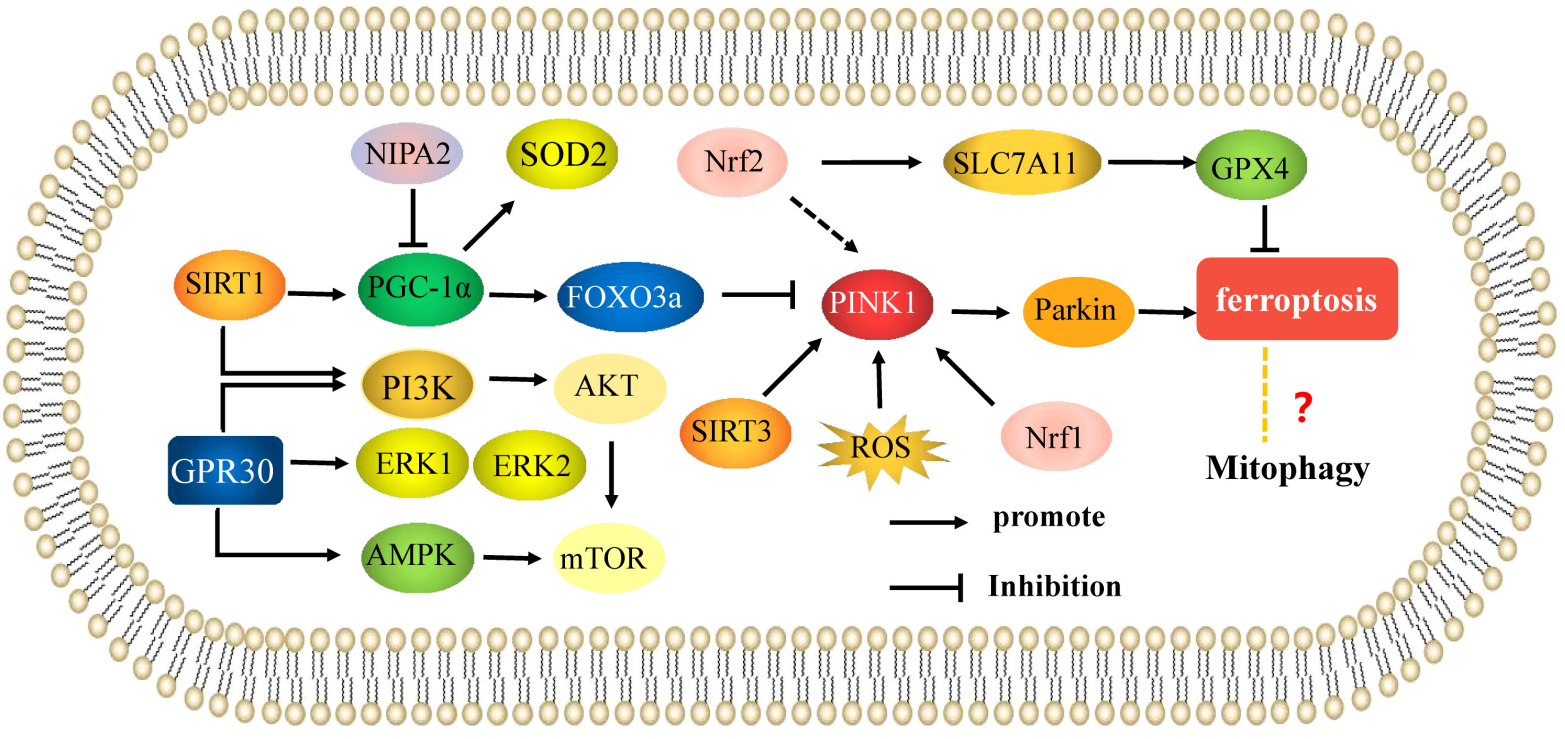
Mitophagy is involved in the mechanisms of the regulation of osteoporosis. Mechanisms of mitophagy regulate osteoblasts and osteoclasts in osteoporosis. The arrows indicate activation and the inhibitory arrows indicate inhibition.

Recent studies have shown that mitophagy is closely related to mammalian target of rapamycin (mTOR) signaling and is involved in the occurrence of OP. Yang established a rat model of osteoporosis using dexamethasone, and found that resveratrol played a protective role in bone loss by promoting mitophagy of osteoblasts. Further studies showed that resveratrol treatment could protect osteoblasts in osteoporotic rats by activating SIRT1-mediated PI3K/AKT/mTOR signaling and enhancing mitophagy. mTOR signaling is also involved in the regulation of mitophagy by 17β-estradiol (63). G protein-coupled receptor 30 (GPR30), also known as G protein-coupled estrogen receptor (GPER), is an estrogen response receptor that mediates estrogen levels. 17β-estradiol has been shown to promote expression of GPR30, activate adenosine 5’-monophosphate (AMP)-activated protein kinase(AMPK)/mTOR signaling by acting on GPR30, and induce mitophagy in osteoblasts (64). Zhang (65) also found that 17β-estradiol could activate PI3K/Akt/mTOR signaling by regulating GPER and inhibit mitophagy in mouse MC3T3-E1 osteoblasts, indicating a protective role in osteoblasts. Therefore, targeting mTOR signaling to enhance mitophagy may have promising effects in the treatment of OP and other bone metabolic diseases. Sun (66) found that the optimal concentration of 17β-estradiol in osteoblasts to induce GPR30 expression was 10^-7^ M, leading to the accumulation of mitophagosomes. In mouse osteoblasts cultured *in vitro*, 17β-estradiol binds GPR30 through the ERK1/2 pathway and promotes mitophagy. These results indicate that 17β-estradiol also protects mouse osteoblasts by enhancing mitophagy through the GPR30-ERK1/2 pathway. GPR30 is an important target of 17 β-estradiol in OP (**Figure 3**).

## 3 Potential therapeutic targeting of mitochondria in osteoporosis

A deeper understanding of mitochondria-related targets and targeted therapies will be important in the treatment of OP. Recent studies have shown that the effects of drugs on mitochondrial function of osteoblasts are mainly focused on SIRT1/3 and Nrf2. Ma (67) found that sodium hydrosulfide (NaHS) could reverse dexamethasone-induced mitochondrial dysfunction by upregulating the expression of SIRT1 and its downstream effector PGC-1α, and SIRT1 silencing abolished the protective effect of NaHS on mitochondrial dysfunction in osteoblasts. In addition, resveratrol can protect osteoblasts in osteoporosis rats by activating SIRT1 target to enhance mitochondrial biogenesis and mitophagy (27, 63) ^75^. Li (68) found that iristein decreased mitochondrial membrane potential, intracellular ROS level, mitochondrial O_2_·^−^ state, ATP production, complex I and IV activity through SIRT3 pathway, indicating that iristein regulated mitophagy and mitochondrial biogenesis in a SIRT3 dependent manner to reverse the AGE induced inhibition of adipocyte stem cell (ASC) osteoblastic differentiation. Yang (69) found that metformin improved mitochondrial function by increasing expression of SIRT3 to prevent H_2_O_2_-induced osteoblast apoptosis and reverse bone loss in ovary-removed mice. Further experimental results showed that metformin increased the expression of SIRT3 through PI3K/AKT signaling, thus reversing H_2_O_2_-induced osteoblast apoptosis. Proanthocyanidins (PACs) are a group of antioxidant polyphenolic flavonoids. Studies have shown that PACs increase Nrf2 nuclear translocation and downstream HO-1 expression levels, restore mitochondrial potential and mtROS, and promote expression of RUNX2, COL1A1, and OCN. Thus, PACs reduce oxidative stress and mitochondrial dysfunction by activating the Nrf2 pathway to protect osteoblast function and inhibit development of glucocorticoid-induced osteoporosis (70). Zhang (71) also found that PACs significantly increased the level of MMP and respiratory chain complex IV activity in osteoblasts, and decreased ROS and mitochondrial superoxide production, indicating that PACs could significantly improve mitochondrial dysfunction and promote osteoblast generation. These results suggest that affecting mitochondrial function by acting on SIRT1/3 and Nrf2 targets may be a potential therapeutic approach for OP.

Ferutin is a natural non-steroidal phytoestrogen. It is an estrogen receptor agonist, which can reduce the level of MMP, ATP and ROS, increase the expression level of PINK1 and Parkin, induce mitophagy to promote osteoblastic differentiation (72). Chen (73) found that vitamin K2 can promote the expression of LC3-II, PINK1 and Parkin proteins to enhance mitophagy in osteoblast, thus playing an anti-osteoporosis role. Studies have found that epigallocatechin-3-gallate (EGCG) is a rich polyphenol in green tea, which can reduce the production of intracellular and mitochondrial ROS, increase the production of ATP, significantly reduce the mitochondrial membrane potential and the expression of mitochondrial autophagy related molecules PINK1 and Parkin, reduce mitophagy, and inhibit osteoclast differentiation (74). These findings indicate that many mitophagy enhancers have been proved to promote osteoblast differentiation, while in osteoclasts, mitophagy inhibitors can treat osteoporosis by inhibiting its differentiation. Therefore, mitophagy regulators are expected to become an effective way to treat OP by maintaining mitochondrial function.

**Figure 4:**
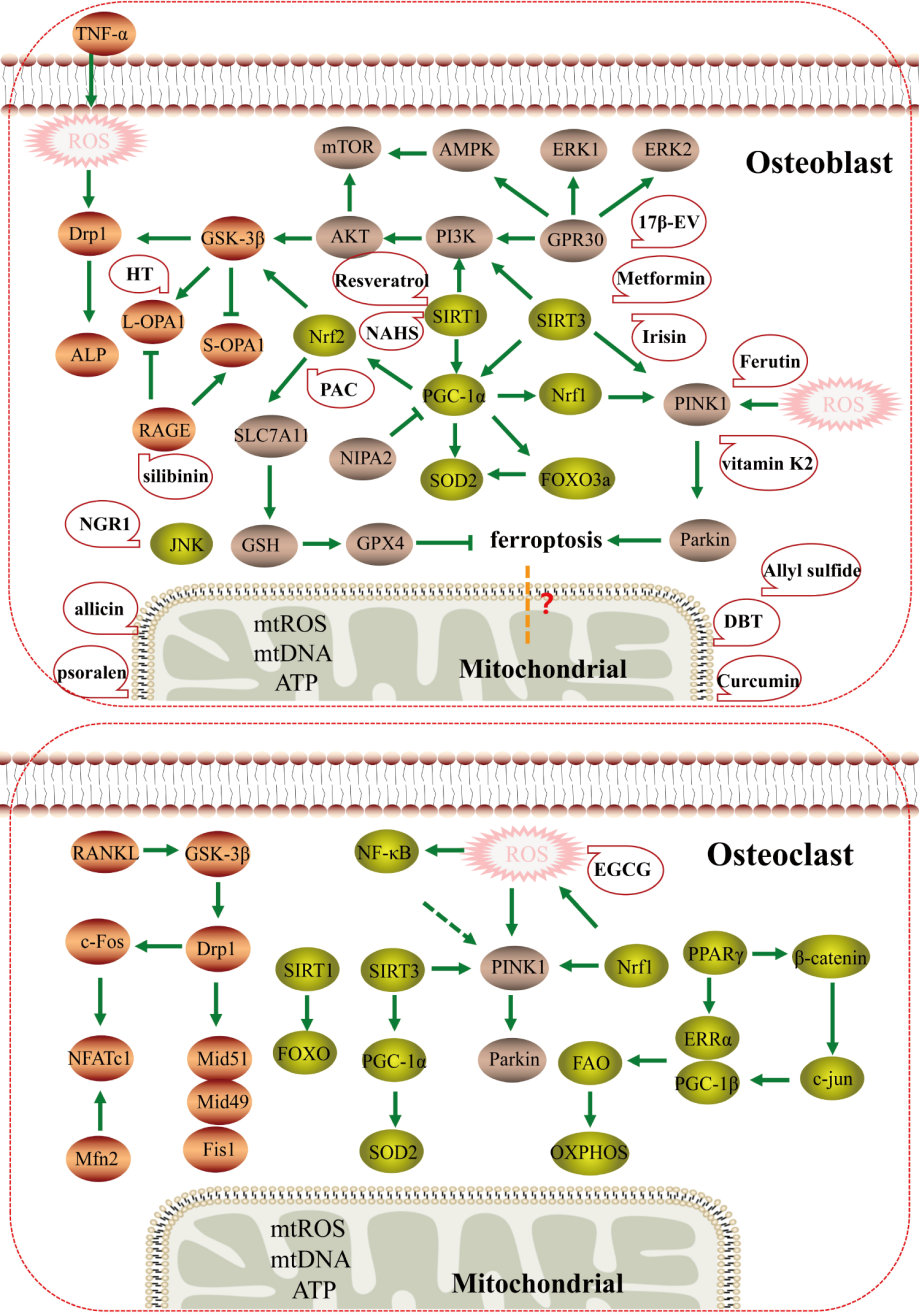
The mechanism and potential targeted therapy of mitochondria in osteoporosis. Mechanisms of mitochondrial dysfunction regulating osteoblasts and osteoclasts in osteoporosis; Mitochondrial dysfunction may have potential relationship with ferroptosis, and traditional Chinese medicine and natural medicine targeting mitochondria may have therapeutic effects.

The traditional Chinese medicine compound Danggui Buxue Tang (*Astragalus membranaceus* and *Angelica sinensis*) has also been shown to increase mitochondrial potential, and cause an increase in mitochondrial length, crista area, total mitochondrial area, area per mitochondrial, and total cell area by approximately 200%, 500%, 180%, and 200%, respectively. It has been shown to regulate mitochondrial function and promote the generation of osteoblasts by influencing mitochondrial morphology and mitochondrial dynamics (75). Traditional Chinese medicine monomers also play an important role in regulating mitochondrial function. Curcumin, a natural antioxidant isolated from turmeric, has a high protective effect on osteoporosis (76). Curcumin can reduce mitochondrial ROS, increase complex III and CoQ activity, and increase mitochondrial membrane potential in osteoblasts, possibly by reducing mitochondrial dysfunction by activating AKT/GSK-3β signaling and attenuating osteoblast apoptosis (77). Therefore, curcumin can significantly reduce oxidative stress-induced mitochondrial dysfunction to inhibit osteoblast apoptosis. Psoralen is a natural flavonoid extracted from the bean psoralea fruit, which is a kind of Chinese herbal medicine for the treatment of OP (78). Psoralen has been shown to promote MMP in osteoblasts, to enhance mitochondrial function during osteogenesis; MMP inhibitors can block the induction of ALP activity and calcium deposition by psoralen in osteoblast culture, indicating that the osteogenic effect of psoralen is closely related to enhanced mitochondrial function (79). Li (80) found that notoginseng saponin R1 (NGR1), an active component in the traditional Chinese medicine notoginseng, reduced expression of c-Jun N-terminal kinase (JNK) and P-JNK, increased mitochondrial membrane potential, mtROS, ATP, and mtDNA, and promoted the levels and activities of ALP, OCN, COLI, and Runx2. Thus, NGR1 can reduce oxidative stress-induced mitochondrial dysfunction and restore osteoblast function by blocking JNK signaling. Therefore, Chinese herbal medicine may be beneficial for the mitochondrial function of osteoblasts. Other studies have found that silibinin, the main flavonoid of silymarin, has strong antioxidant and mitochondrial protective properties. In osteoblasts, it can directly downregulate the expression of RAGE, maintain the balance of L-OPA1 and S-OPA1, reduce mitochondrial oxidative stress and mitochondrial membrane potential, and significantly increase ATP production. Changes in mitochondrial morphology and mitochondrial dynamics can protect osteoblasts from apoptosis (23). Hydroxytyrosol (HT), an important compound in virgin olive oil, also reduces rapid cleavage of L-OPA1 to S-OPA1 in osteoblasts by regulating the AKT/GSK-3β pathway, reduces its mitochondrial dysfunction, and inhibits osteoblast apoptosis (22). Ding (81) found that allicin, a natural component of garlic, could improve mitochondrial function to inhibit oxidative stress-induced osteoblast apoptosis, possibly *via* activation of PI3K/AKT and CREB/ERK signaling. Allylsulfur, a garlic-derived organosulfur compound, attenuates mitochondrial dysfunction caused by superoxide production, mitochondrial biogenesis, and energy metabolism, and prevents mtDNA release to promote osteogenic differentiation, mineralization, and increased bone mineral density of bone marrow mesenchymal stem cells (82) (**Figure 4**).

The above studies confirmed that the regulatory mechanism of mitochondrial regulators on bone metabolism plays a role in osteoporosis mainly through changes in mitochondrial dynamics, mitochondrial biogenesis, and mitochondrial autophagy. These results indicate that mitochondria are important targets for osteoprotection and that targeted therapy of mitochondria by traditional Chinese medicine and other natural drugs may also be an important focus for treatment of OP in the future. However, most of the current research on mitochondrial targeted therapy has focused on osteoblasts, and drugs that inhibit mitochondria in osteoclasts still need to be further explored.

## 4 Conclusions and perspectives

**Figure 5:**
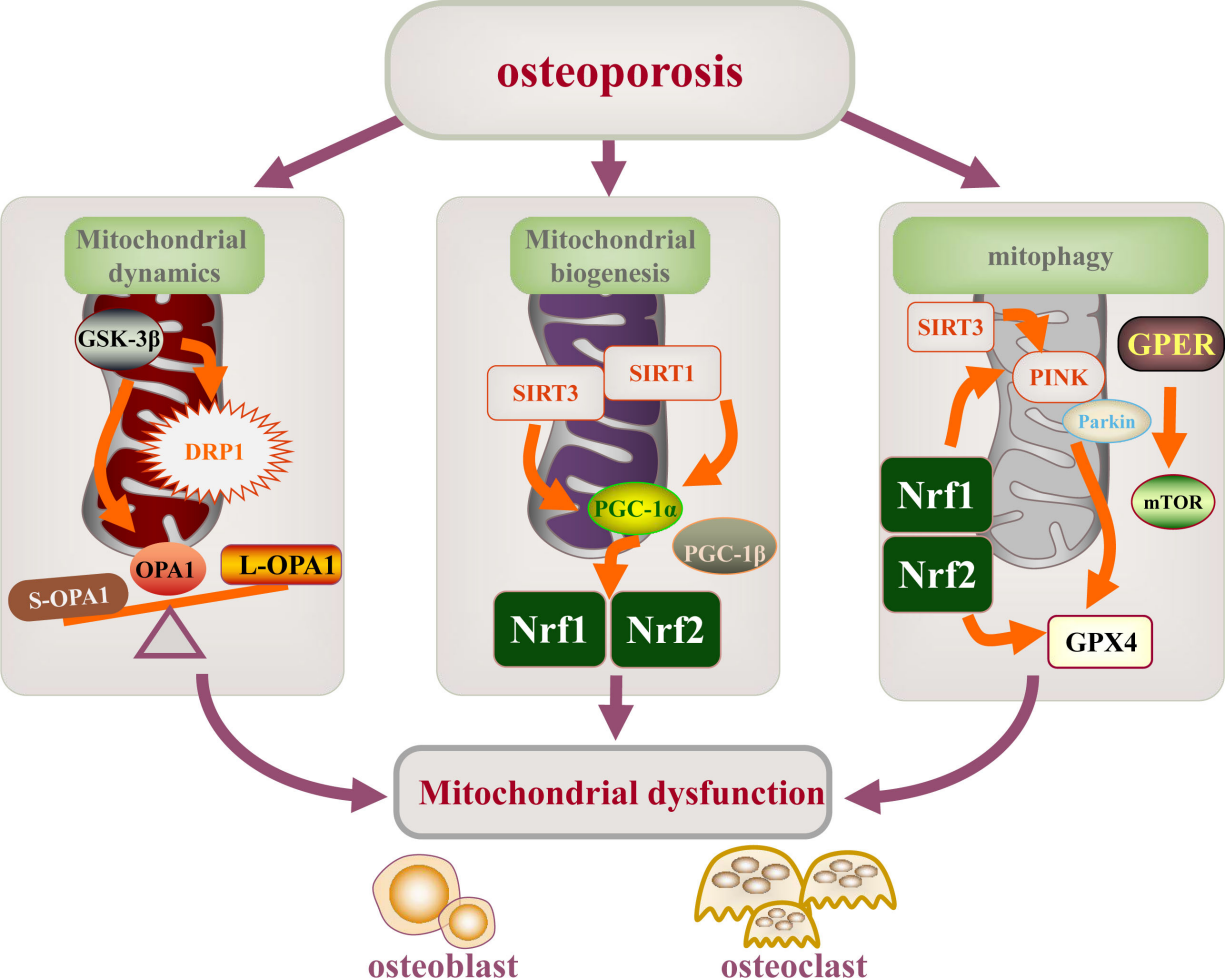
Mitochondrial dysfunction is involved in the mechanism of osteoporosis. Mitochondrial dysfunction (mitochondrial dynamics, mitochondrial biogenesis, and mitophagy) leading to the development and progression of osteoporosis. The main targets of mitochondrial dysfunction are DRP1, OPA1, GSK-3β, mTOR, SIRT1/3, PGC-1α, NRF2/2, PINK1, and Parkin.

In this review, we endeavored to summarize the pathological mechanism that mitochondrial quality control is involved in the occurrence and progression of OP (diabetes induced osteoporosis and postmenopausal osteoporosis), mainly the target, signal pathway and targeted treatment of mitochondrial function *in vitro* and *in vivo* models of osteoporosis, in which there are abnormal mitochondrial dysfunction mechanisms in OP, including dynamic changes, mitochondrial biogenesis and mitophagy. The important contribution of abnormal mitochondrial function regulation mechanism to the occurrence and development of osteoblasts, osteoclasts and osteoporosis has been fully confirmed. Recent studies on the regulation mechanism of mitochondrial function mainly have focused on the targets of DRP1, OPA1, GSK-3β, mTOR, SIRT1/3, PGC-1α, Nrf1/2, PINK1, and Parkin, which regulate differentiation, apoptosis, and activity of osteoblasts and osteoclasts by mediating mitochondrial dysfunction (**Figure 5**). Meanwhile, numerous drugs have been found to regulate the function of osteoblast or osteoclast mitochondria by regulating the expression of these targets to treat OP.

Mitochondrial metabolic disorders and dysfunction have shown increasing value for medical application in many chronic diseases, and are now considered to be an important target for the treatment of chronic diseases (83). As a chronic bone disease, osteoporosis mainly includes diabetes induced osteoporosis and postmenopausal osteoporosis. diabetes induced osteoporosis is due to insulin deficiency and endocrine dysfunction leading to bone density decline and bone microstructure changes, while postmenopausal osteoporosis is bone loss caused by estrogen deficiency. The common pathogenesis of the two types of osteoporosis is that bone formation mediated by osteoblasts is less than bone resorption mediated by osteoclasts, resulting in an imbalance in bone homeostasis. An increasing number of studies have confirmed that mitochondrial function is essential to improve bone homeostasis (84). Mitochondrial quality control is of great significance for homeostasis of the mitochondrial network and normal mitochondrial function. Further studies have found that traditional Chinese medicine and natural medicines can regulate mitochondrial function by regulating the expression of different targets, and have therapeutic effects on OP, which may provide new insights into the development of mitochondrial regulators of OP.

Undoubtedly, the study of mitochondrial regulation and function will not only provide new insights into disease diagnosis and prognosis but also promote the development of potential therapeutic strategies. It is interesting to note that the mitochondrial functions of targets in the pathophysiology of OP are various; these mechanisms may offer multiple targets or pathways. The challenge remains with regard to how to form a specific regulation network to influence the metabolism of osteogenesis, and osteoclasts still need further discussion. This will also be a focus in further study of the pathogenesis of OP. In recent years, ferroptosis has been shown to be a key mechanism regulating bone formation and resorption, and mitochondrial function can regulate ferroptosis. However, the specific regulatory role relating these two functions in OP has not been thoroughly investigated. In terms of mitochondrial therapeutic targets, although some traditional Chinese and natural medicines have been shown to influence the occurrence and development of OP *via* mitochondrial regulation, research of these has been primarily confined to cell and animal model experiments; effects of these drugs on the human body with OP will require further investigation. At present, there are relatively few studies on the targets and targeted therapy of mitochondrial function in OP. With further exploration, mitochondria-related mechanisms may become novel drug targets for the prevention and treatment of OP, which will provide a scientific basis and new ideas for the precise treatment of OP.

## Author contributions

CY and YS were responsible for the collection, analysis of the references, and wrote the manuscript and are considered as co-first authors; XL and FA contributed to the conception of the study, the submission and the revision of the manuscript and are considered as co-corresponding author; LY, DL, BS, and JW helped in drawing the figures of the manuscript and checking the manuscript. All authors contributed to the article and approved the submitted version.

## Glossary

**Table:**

ATP	adenosine triphosphate
DRP1	dynamic-related protein 1
Mff	mitochondrial fission factor
Fis1	mitochondrial fission protein 1
MiD49	mitochondrial dynamics protein of 49 kDa
MiD51	mitochondrial dynamics protein of 51 kDa
Mfn1/2	Mitofusion 1/2
OPA1	optic atrophy 1
ROS	reactive oxygen species
ALP	alkaline phosphatase
TNF-α	tumor necrosis factor-α
RANKL	receptor activator of NF-κB ligand
GSK3β	glycogen synthase kinase-3β
NFATc1	nuclear factor 1 of activated T-cells
mtROS	mitochondrial ROS
siRNA	interfering RNA
AGEs	advanced glycation end products
mtDNA	mitochondrial DNA
PGC-1α/β	peroxisome proliferator-activated receptor-gamma coactivator-1α/β
SIRT1/3	Silencing information regulator 1/3
Nrf1/2	nuclear respiratory factor 1/2
ERRα	estrogen related receptor α
TFAM	mitochondrial transcription factor A
OCN	osteocalcin
OPN	osteopontin
Runx2	runt-related transcription factor 2
NaB	Sodium butyrate
FoxO3	aperacetylate forkhead box protein O3 a
SOD2	manganese superoxide dismutase
HO-1	gene heme oxygenase 1
COL1A1	collagen type 1 alpha1
OSX	Osterix
mtTFA	mitochondrial transcription factor A
PPARγ	peroxisome proliferators-activated receptors
β-FAO	fatty acid β-oxidation
OXPHOS	oxidative phosphorylation
CTX-1	C-telopeptide of type 1 collagen
TRAP	tartrate-resistant acid phosphase
MMP-9	matrix metalloprotein-9
CTSK	recombinant cathepsin K
PINK1	Pten-induced putative kinase 1
Parkin	Parkinson disease-related gene
BSP	bone sialoprotein
NIPA2	Prader-Willi/Angelman syndrome region protein 2
MMP	mitochondrial membrane potential
NF-κB	nuclear factor-κB
FtMt	Mitochondrial ferritin
CCCP	carboacylcyanom-chlorobenzene hydrazide
SLC7A11	solute carrier protein 7 family member 11
GPX4	glutathione peroxidase 4
GSH	glutathione
PI3K	phosphatidylinositol 3-kinase
AKT	protein kinase B
mTOR	mammalian target of rapamycin
AMPK	adenosine 5’-monophosphate (AMP)-activated protein kinase
GPR30	G protein-coupled receptor 30
GPER	G protein-coupled estrogen receptor
NaHS	sodium hydrosulfide
ASC	adipocyte stem cell
PACs	Proanthocyanidins
EGCG	epigallocatechin-3-gallate
NGR1	notoginseng saponin R1
JNK	c-Jun N-terminal kinase
HT	Hydroxytyrosol
